# Wheat in 2025: challenges for sustainable production and consumption

**DOI:** 10.1093/jxb/eraf514

**Published:** 2025-11-21

**Authors:** Peter R Shewry

**Affiliations:** Rothamsted Research, Harpenden AL5 2JQ, UK; University of Reading, Earley, Reading RG6 6BZ, UK; Max-Planck-Institut für Molekulare Pflanzenphysiologie, Germany

**Keywords:** Adverse reactions, human health, human nutrition, nitrogen fertilization, sustainability, wheat

## Abstract

Wheat is the dominant food crop in its contribution to global nutrition, and production has, and may continue to, increase in line with the increase in the global population. However, the production of wheat for breadmaking in countries with high input systems (notably in Western Europe) is highly dependent on nitrogen fertilization in order to produce grain with high protein content, raising concerns about sustainability and adverse impacts on the environment. In addition, the consumption of wheat is decreasing in some countries due to concerns about adverse effects of wheat, and particularly gluten, on health. The scientific basis for these concerns is discussed and strategies proposed to reduce nitrogen inputs for breadmaking and address the concerns of consumers.

## Teaser

Challenges to the production and consumption of wheat due to the requirement for nitrogen fertilization and perceived adverse impacts on health are discussed in relation to its global role in nutrition.

## Introduction: wheat production and consumption

**Fig. 1.**
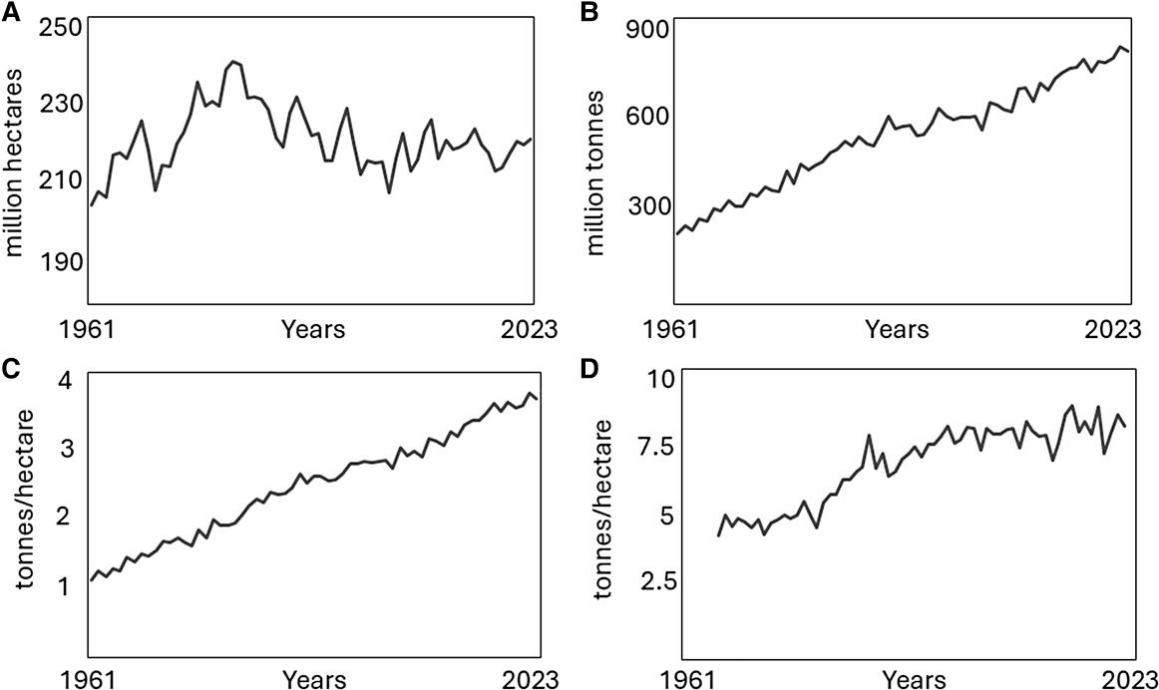
Area (A), volume (B), and yield (C) of wheat produced globally and yield of wheat produced in the UK (D), during the period 1961 to 2023. Data from FAOSTAT (2025).

The dominance of wheat in global agriculture has increased over the past few decades and it is currently the major staple food crop in the world, based on production, consumption, and contribution to human nutrition and health. Global production continues to increase, from 6.84 million tonnes in 2009 to 8.11 million tonnes in 2023, and the increased production over the past 60 years has been achieved without an increase in the area under cultivation, by increasing yields through a combination of genetic enhancement and improved agronomy (Fig. 1A, B).

Since the average global yield (3.6–3.7 t ha^–1^) of wheat (Fig. 1C) is well below the average yields achieved in intensive production systems in Western Europe (e.g. about 8 t ha^–1^ in the UK, Fig. 1D), and these are in turn well below the maximum yields which have been achieved in the same countries (up to 16 t ha^–1^), there is no reason why the average global yield, and hence total global production, of wheat should not continue to increase.

The global consumption of wheat has also continued to increase, particularly in countries which are undergoing urbanization where it is associated with the adoption of a ‘Western’ lifestyle. These include countries in which the climate is not suited to high volume wheat production, including much of Sub-Saharan Africa, resulting in the displacement of traditional crops and foods (Mattei *et al*., 2015).

The importance of wheat in global food security has been recognized by massive investments in basic research to underpin wheat improvement, including the detailed characterization (including genome sequencing) of a diverse range of germplasm, providing variation in traits of interest to wheat researchers as well as breeders (see, e.g. Cheng *et al*., 2024; Jiao *et al*., 2024).

### Challenges and limitations to wheat production

Bread wheat originated in the Fertile Crescent region of South-West Asia about 10 000 Before Present (BP) and then spread to all continents: to Western Europe by about 7000 BP, to China (3000 BP), Africa (Egypt, 8000 BP), the Americas (Mexico, 1529 CE), and Australia (1788 CE) (Feldman, 2001). During this migration wheat was exposed to new environments and responded by adaptation resulting in thousands of genotypes, some of which are now preserved in gene banks. This adaptation underpins the wide geographical range of wheat production, from 67°N in Scandinavia and Russia to 45°S in Argentina, including elevated regions in the tropics and sub-tropics (Feldman *et al*., 1995). However, the high demand for wheat means that it is sometimes grown in less suitable climates resulting in very low yields: FAOSTAT (2025) reports wheat yield data for 2023 from 123 countries, with 12 of these (mainly located in Sub-Saharan Africa and the Middle East) reporting yields of 1 ha^–1^ or less.

In global terms, water availability is biggest single factor determining wheat yield (Langridge *et al*., 2022), with drought often being associated with heat stress. Langridge *et al*. (2022) note that about half of the wheat grown globally experiences heat stress with 20 million ha experiencing water deficits. A substantial proportion of global wheat production is irrigated, meaning that it is susceptible to depletion of water sources. For example, Ai *et al.* (2024) calculated that irrigation accounted for 37% of the production of wheat in the 20 major wheat-producing countries (which together accounted for 86% of total global production).

The challenges of climate change and water depletion face many major crops, together with environmentally sustainable control of pests and pathogens and reduced availability of land due to degradation and urbanization. However, wheat production faces additional challenges related to its role in food processing and human nutrition and health.

Given sufficient water and light and acceptable temperatures the yield of wheat is determined primarily by nitrogen availability, particularly the application of nitrogen-containing fertilizers which drive the high yields achieved in Western Europe and other countries with high input production systems. However, wheat differs from other major crops in that nitrogen is required not only to achieve high yields but also high contents of the major grain storage proteins (gluten) which determine the quality of the grain for processing, including breadmaking. The requirement for high protein grain for breadmaking poses a significant challenge in the high production systems used in traditional wheat-consuming areas of Western Europe, North America, and Australia as high yields result in dilution of grain protein with starch, requiring higher levels of nitrogen application than are optimal for grain yield.

There are also concerns in some traditional wheat-consuming areas about the role of wheat, and of wheat proteins in particular, in triggering specific adverse effects on health, leading to increasing adoption of wheat-free or gluten-free diets. These two challenges are therefore discussed below.

## Increasing wheat production while reducing the reliance on nitrogen inputs

As already discussed, the increases in global wheat production (Fig. 1B) result from increased yields which in turn result from two factors: increases in the yield potential of the crop and improved agronomy. Although it been suggested that scientific advances could lead to step change increases in yield potential, for example, it has been estimated that a 37% increase could result from improving canopy photosynthesis alone (Guarin *et al*., 2022), global improvements continue to result from small increments and by conventional breeding supported by modern genetics (including genomic selection) rather than biotech approaches.

However, whereas the global yield of wheat continues to increase, the yield in the UK has been stable at about 8 t ha^–1^ for over 20 years (Fig. 1D). The 5-year mean (2019–2023) yield in the UK was 8.1 t ha^–1^, with lower mean yields in the two major Western European wheat producing countries, 7.1 t ha^–1^ in France and 7.5 ha^–1^ in Germany, with only New Zealand averaging above 9 t ha^–1^ (9.5 t ha^–1^).

These yields are significantly below the yield potentials of modern cultivars. For example, in the UK the yields of the ‘control’ cultivars used by the Agricultural and Horticultural Development Board (AHDB) to evaluate new cultivars in multisite field trials ranged between 10.8 and 11.6 t ha^–1^ over the period 2021–2025 (AHDB, 2025). (Recommended Lists for cereals and oilseeds (RL) | AHDB).

The failure of farmers to achieve the yields observed in experimental field trials results from a number of factors, including the use of sub-optimal sites (in terms of soil type and environmental conditions). However, the major factor limiting yields in many systems is restricted use of agrochemicals, chiefly nitrogen fertilizer, due to environmental considerations or economic factors.

### Nitrogen fertilizer drives grain yield

It has been estimated that ‘the number of humans supported per hectare of arable land has increased from 1.9 to 4.3 persons between 1908 and 2008 and suggested that this was mainly made possible by the availability of nitrogen fertilizers derived from the conversion of atmospheric nitrogen to ammonia by the Haber–Bosch process (Erisman *et al.*, 2008) which was first used on an industrial scale in 1913. Ammonia production is energy intensive, accounting for 1% of global energy consumption and 1.4% of carbon dioxide emissions (Capdevila-Cortada, 2019). Nitrogen fertilizer is often the major input cost for farmers, particularly in the UK, while fertilizer which is not absorbed by the crop may have a negative environmental footprint. Hence, although it is clearly not possible to conceive of a world without fertilizers, it is important to optimize their use to maximize benefits and reduce adverse impacts.

Nitrogen fertilization is important for grain yield as it supports the development of a vigorous canopy and efficient photosynthetic apparatus, with Rubisco alone accounting for 25–30% of the total nitrogen in leaves (Evans, 1989). However, nitrogen is also required for seed development, to synthesize structural and metabolic proteins and the gluten storage proteins which are crucial for processing of wheat.

### Grain protein determines processing quality

Wheat is unique in that doughs made from milled grain exhibit an unusual combination of physical properties, a combination of viscosity, elasticity, and extensibility, which allow the dough to be processed into a range of foods (notably leavened and unleavened breads, other baked products, pasta and noodles) and to be used as an ingredient in processed foods. These properties are determined by the gluten proteins which are the major group of storage proteins in the starchy endosperm of the grain and generally estimated to account for about 80% of the total grain proteins. However, gluten is a complex mixture of over 50 proteins which are classically divided into two groups: the polymeric glutenins which contribute dough strength (elasticity) and the monomeric gliadins which contribute mainly to cohesion and extensibility. The structures and properties of gluten proteins have been widely studied and reviewed (Shewry *et al*., 2009; Shewry and Belton, 2024).

The protein content of wheat grown in temperate climates, such as the UK, generally ranges between about 10% and 15%, although it varies more widely (from about 7–20%) in more extreme environments. Milling of the grain separates the starchy endosperm storage tissue from the more protein-rich aleurone and embryo (germ) tissues resulting in a lower protein content of white flour compared to wholegrain. This reduction is generally by about 2%. For example, a comparison of a doubled haploid population of 168 lines grown in three environments in China showed mean protein contents of 13.89%, 13.64%, and 13.14% in wholegrain and 11.78%, 11.12%, and 10.88% in white flour (at 70% flour extraction rate) (Zhao *et al*., 2010). However, because the gluten proteins are only present in the starchy endosperm their concentrations are higher in white flour than in wholegrain.

The requirements for protein content and gluten properties vary between different processes. Stronger doughs with higher protein contents are generally required for making bread than for cakes and biscuits where lower protein contents and more extensible doughs are required. However, there is variation within types of products, for example, French baguettes require lower protein content than sliced sandwich breads. Furthermore, the lower contents of gluten in wholemeal flours means that higher grain protein content is required when processing wholemeal flours than with white flours.

### Reducing the nitrogen requirement for breadmaking wheat

**Fig. 2.**
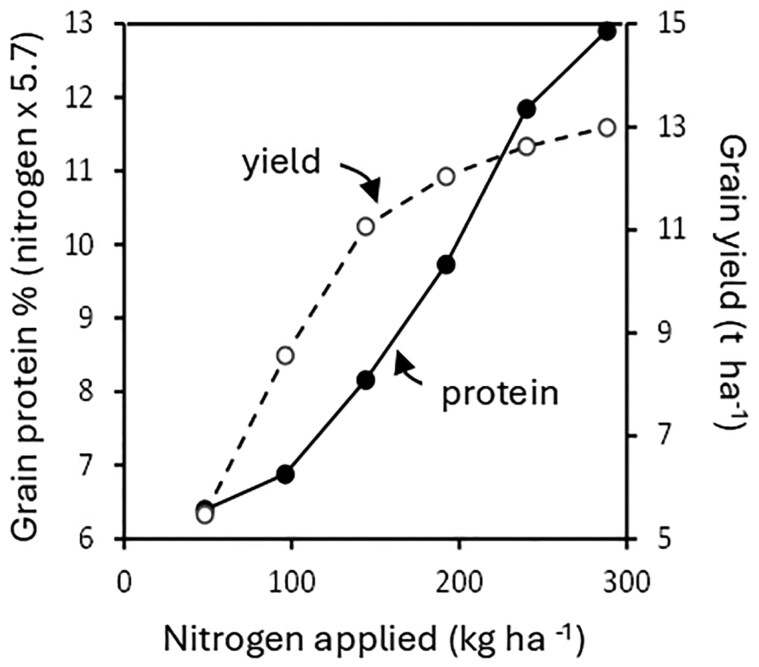
Relationship between application of nitrogen fertilizer, grain yield, and grain protein content (calculated as grain nitrogen ×5.7) for breadmaking wheat cv. Crusoe grown as first wheat in the Broadbalk long term experiment at Rothamsted Research in 2014. Yield is expressed on an 85% dry weight basis and protein content on a dry weight basis. The data were kindly provided by the Lawes Agricultural Trust and Rothamsted Research (e-RA database). Taken from Gooding and Shewry (2022) with permission.

There is a clear relationship between nitrogen ferilization, total grain protein content (Fig. 2) and the proportion of gluten proteins. For example, comparisons of four cultivars grown in the UK showed 72.19–73.85% and 78.81–81.83% gluten proteins as % total proteins in wholemeal flours of grains grown with low and high levels of nitrogen fertilization, respectively (He *et al*., 2013). This effect is exploited by farmers who vary the levels of nitrogen applied to achieve the required protein content. Consequently, higher levels of fertilizers are applied to breadmaking wheats compared to cultivars grown for livestock feed or fermentation.

However, the production of wheat for breadmaking may require levels of nitrogen application which are above those which are optimal for yield. This is illustrated by Fig. 2 which shows data from the Broadbalk long term wheat nutrition experiment at Rothamsted. Whereas the effect of nitrogen on yield decreased above 200 kg ha^–1^, about 300 kg ha^–1^ was required to meet the protein required for breadmaking wheat in the UK (13% on an 14% moisture basis, corresponding to about 15% dry weight). Closing this difference, to allow the production of breadmaking wheat at the optimum fertilizer application for yield, is an important target to improve the sustainability of wheat production.

Many studies have been aimed at reducing the nitrogen requirement for producing breadmaking wheat and it is important to consider these in the context of the minimum requirements for processing. The minimum grain protein content of 13% required in the UK corresponds to a flour protein content of about 11%. Assuming a grain yield of 10 t ha^–1^, this corresponds to 1.3 tonnes of protein per hectare, which contains 228 kg nitrogen (assuming a conversion factor of N×5.7=protein). Hence, 228 kg N is the minimum amount that is required to sustain the required grain protein content and yield, assuming 100% nitrogen recovery in the grain.

Bearing the caveats above in mind, how can we decrease the nitrogen requirement for breadmaking wheat? The first option is to increase our ability to make acceptable bread from low protein flour. The required protein content actually varies between processes and products (as discussed above) and the Chorleywood Bread Process (CBP), which is dominant in the UK and some other countries (including Australia, New Zealand, and South Africa), actually allows the use of flour with up to 1% less protein than traditional breadmaking systems. Increasing dough elasticity by genetic improvement may also allow the use of lower protein flours (Shewry *et al*., 2023b).

However, a major factor determining the minimum protein requirement is the need to allow for variation in quality due to the effects of the environment. This is because a higher protein content can, to some extent, compensate for lower protein quality. Environmental impacts on quality have become an increasing concern in the changing climate, and can affect global markets. For example, variation in the quality of the UK wheat harvest resulted in the volume of milling wheat imported from the European Union increasing from 295 814 tonnes in 2022–23 to 570 595 tonnes in 2023–24, representing 6.1% and 10.47% of the total grist (grain used for milling), respectively (data provided by UK Flour Millers). Although the UK food system is able to cope with such fluctuations without effects on food prices, this may not be the case for other countries.

Consequently, strategies to maintain grain protein content at lower nitrogen input are being explored. The most promising of these is to exploit genetic variation in grain protein deviation (GPD).

Many studies have shown that grain protein content is inversely correlated with grain yield and hence modern high yielding varieties generally have lower protein contents than older types (Shewry *et al*., 2016). However, Monaghan *et al*. (2001) showed that some genotypes deviated positively or negatively from the simple regression line which could be calculated for grain yield and protein content when collections of genotypes were compared and introduced the term grain protein deviation (GPD). Positive GPD represents greater efficiency in transferring nitrogen into the developing grain and has moderate heritability [reported as 0.44 by Mosleth *et al*. (2020) and 0.53 by Richard *et al*. (2025)]. Although GPD is under multigenic control, with some quantitative trait loci (QTLs) overlapping with those for grain protein content, several major QTLs (e.g. on chromosomes 3B and 5B) are emerging as candidates for exploitation by breeders and elucidation of mechanisms (see Richard *et al.*, 2025).

### Potential for exploiting biological nitrogen fixation

Developing symbioses between nitrogen-fixing bacteria (diazotrophs) and cereals (including wheat) has been a target for crop improvement for over 30 years, building on our detailed understanding of the molecular mechanisms underpinning nitrogen-fixing root nodules in legumes.

The most ambitious approach is to develop rood nodules in cereals through engineering key genes identified as involved in nodule development and nitrogen fixation in legumes. This approach has so far failed to produce functional nodules (Pankievicz *et al*., 2019; Tajima *et al*., 2025), which is perhaps not surprising in view on the complexity of the system.

A more promising and less technically-demanding approach is to engineer wheat plants to secrete chemicals which induce the colonization of the root surface by diazotrophs, resulting in the formation of nitrogen-fixing biofilms. Tajima *et al.* (2025) have recently used this approach, by editing multiple genes in the flavonoid pathway of wheat to secrete apigenin (4′,5,7-trihydroxyflavone), a flavone present in many fruits and vegetables. Growth of the plants hydroponically in the presence of the diazotroph *Azospirillum brasilense* showed the formation of biofilms containing the bacterium while the edited wheat exhibited increased nitrogen content, improved photosynthetic performance, and higher grain yield relative to wild-type controls when grown in pots under nitrogen-limiting conditions. These results indicate that the approach could be used to develop new types of wheat which require less nitrogen fertilization. However, several possible limitations need to be considered. Firstly, the approach requires sophisticated editing of multiple genes which may pose challenges for affordability and acceptability. Secondly, the effectiveness of the approach under high input systems needs to be demonstrated as the diversion of nutrients to the roots could result in a yield penalty.

## Impacts of wheat on nutrition, health, and disease

Whereas the global consumption of wheat continues to rise, particularly in countries where it has not been traditionally grown and consumed, the consumption is either static or decreasing in some countries that traditionally consume wheat, notably in Europe, North America, and Australia. For example, Lockyer and Spiro (2020) reported that total UK house purchases of bread decreased from 1019 to 527 g person^–1^ between 1974 and 2017/8. These decreases partly reflect increasing prosperity, with staple foods being displaced by more varied diets including greater consumption of meat, and may also reflect increasingly multicultural populations consuming more diverse diets. However, they also reflect concerns about the role of wheat in health and disease, including the role of highly refined foods in increasing the risk of non-communicable diseases associated with the ‘Western diet and lifestyle’ (obesity, type 2 diabetes, cardio-vascular disease) and specific adverse responses to wheat or gluten (notably coeliac disease and non-coeliac wheat sensitivity, NCWS).

We therefore need to consider two questions: are the concerns supported by scientific studies and, if so, how can we reduce their impacts?

### Wheat and health

The contribution of wheat, and particularly wheat consumed as bread, to dietary intakes of macro- and micro-nutrients has been extensively studied. Wheat is particularly important as a source of energy, providing about 20% of the total intake of calories globally and up to half in some countries. However, it is also a major source of essential and beneficial nutrients, notably protein, dietary fibre, B vitamins, minerals (notably Fe, Zn, and Se), and phytochemicals (notably phenolic acids) with proposed health benefits (see Shewry and Hey, 2019; Weegels, 2019; Laskowski *et al*., 2019; Lockyer and Spiro, 2020). The contribution of wheat to the intake of dietary fibre is particularly important in Western diets as the consumption of fibre is associated with reduced risk of a range of chronic diseases including cardiovascular disease, obesity, type 2 diabetes, and colorectal cancer (Aune *et al*., 2016; Veronese *et al*., 2018; Barrett *et al*., 2019; Guo *et al*., 2021). For example, breads contribute about 8–10% of the total fibre intake of UK adults (Steer *et al*., 2008; Roberts *et al*., 2018). However, the daily intake of fibre in many countries is below dietary recommendations (Lovegrove *et al*., 2025); about 20 g day^–1^ in UK adults compared with a recommended intake of 30 g day^–1^ (Shewry *et al*., 2023a).

Most studies relating the intake of cereal fibre to disease risks have been carried out on wholegrain cereals which have about three times the fibre content (11–15% dry weight) of white flour (about 4–5% dry weight) (Shewry *et al*., 2024). Furthermore, wholegrains also have higher contents of B vitamins, minerals, and phenolics which may contribute to the reduced disease risks.

The fact that wholegrain products have established health benefits often leads to the view that bread and other products made from white flour must be intrinsically unhealthy, and this is also suggested by the classification of factory-produced breads as ‘ultra-processed foods’ (UPFs) based on the widely-used NOVA classification (Monteiro *et al*., 2019).

In fact, published studies provide no evidence that white flour products are intrinsically unhealthy. For example, although it is commonly thought that white bread is more rapidly digested than wholemeal (which is finely milled) and wholegrain breads, leading to a faster increase in blood glucose and increased risk of type 2 diabetes, a meta-analysis showed no statistically significant differences in increases in blood glucose following consumption of white compared with wholemeal products (Musa-Veloso *et al*., 2018). However, it is possible that more coarsely milled wholegrain products are digested more slowly. A large multinational cohort study also showed that although total intake of UPFs was associated with increased risk of cancer-cardiometabolic multimorbidity, the consumption of UP breads and cereals was associated with reduced risk (Cordova *et al*., 2023). Hence, it can be concluded that white bread and other products from white flour are not intrinsically unhealthy but may continue to be consumed in moderation as part of a healthy balanced diet (Shewry *et al*., 2026).

### Adverse reactions to wheat

Although wider concerns about wheat and chronic diseases have contributed to the decline in wheat consumption, the major factor responsible for the adoption of wheat-free or gluten-free diets is a perceived increase in adverse reactions. There is a large and confusing literature on the topic but the reactions can broadly be divided into three types (Table 1).

**Table 1.** Summary of the properties of adverse reactions to wheat: coeliac disease (CD), wheat allergy (WA), and non-coeliac wheat sensitivity (NCWS)

	CD	WA	NCWS
Time interval between exposure and symptoms	Weeks to years	Minutes to hours	Hours to days
Prevalence in adults	Varies between 1% and 2%, global mean about 1.4%	0.2%	Largely self-diagnosed, estimates range from 0.5% to 10% of population. Probably higher than CD.
Pathogenesis	T-cell mediated auto-immunity	IgE mediated allergy	Not fully understood, may involve innate immune system
Triggering substances	Gluten (gliadin and glutenin) proteins, possibly ATIs	ATIs, gluten proteins, other proteins	Not known
Symptoms	Not clearly distinguishable between the three disorders. Common intestinal symptoms include bloating, abdominal pain, diarrhoea, nausea, epigastric pain, and alternating bowel habits. Common extra-intestinal symptoms include lack of well-being, tiredness, headache, anxiety, and foggy mind.		

Modified from Brouns *et al.* (2019) to include data discussed in the text.

Two types of reaction, classical food allergy (wheat allergy, WA) which is mediated by immunoglobulin E (IgE) and coeliac disease (CD) and related T-cell mediated auto-immune responses, are well understood in terms of their prevalence, mechanisms, and triggering substances (Brouns *et al*., 2019). Their combined prevalence in adults is below 2% of the global population, about 1.4% for CD (reviewed by Brouns *et al*., 2019) and 0.2% for WA (Zuidmeer *et al*., 2008). Although the prevalence of diagnosed CD has increased, at least some of this increase has resulted from increased awareness and improved diagnosis (particularly in adults as opposed to children). However, it is estimated that many individuals with CD remain undiagnosed and that this number is increasing (Rubio-Tapia *et al*., 2012).

The third group of reactions is less readily defined and well-understood and was initially termed ‘non-coeliac glutensensitivity’ (NCGS). However, ‘non-coeliac wheat sensitivity’ (NCWS) is now more widely used as there is no evidence that gluten is responsible. NCWS is largely self-diagnosed with a wide range of reported symptoms including gastrointestinal responses similar to those of irritable bowel syndrome (IBS) and wider symptoms such as tiredness, headache, dermatitis, pains in muscles and joints, depression, anxiety, and anaemia. The aetiology is still poorly understood but it may involve activation of the innate immune system. The range of symptoms and the limited understanding of the aetiology pose challenges for diagnosis, but an expert group has recommended a gluten-free diet followed by a double-blind placebo-controlled gluten challenge, with variation of 30% or more in one to three main symptoms being a positive result in both phases (Catassi *et al*., 2015). Estimates of the prevalence vary widely, from less than 1% to 10% of the population (Ludvigsson *et al*., 2013).

### Identification of triggering substances

Two groups of wheat proteins appear to be particularly active in triggering adverse responses. These are gluten proteins and amylase/trypsin inhibitors (ATIs), a group of water-soluble proteins accounting for 2–4% of the total protein (reviewed by Geisslitz *et al*., 2021).

Although coeliac disease was first described in Ancient Greece about 2000 years ago, the role of wheat in triggering the response was only recognised by Samuel Gee working in London in 1888 and the specific role of gluten by Dicke working in the Netherlands in 1950. It has become one of the most intensively studied adverse responses to a plant-based food, leading to the identification of the crucial role of the proline- and glutamine-rich peptides which constitute the central repetitive domains of the gluten proteins (reviewed by Shewry *et al*., 2009). The presence of these two amino acids reduces digestion by proteases in the gastro-intestinal tract generating glutamine-rich peptides which pass between or through the intestinal epithelial cells to reach the lamina propria (a thin layer of connective tissue) where selected glutamine residues are deamidated by the enzyme tissue transglutaminase 2 (TG2). This deamidation increases the affinity of the ‘coeliac epitopes’ to the human leucocyte antigens (HLA)-DQ2 or (HLA)-DQ8, triggering the autoimmune response which is responsible for the symptoms, notably damage to the small intestinal mucosa leading to malabsorption (see the detailed review of Lindfors *et al*., 2019). Currently over 40 coeliac epitopes (each comprising nine amino acid residues) specific for the DQ2 and DQ8 forms of the disease have been identified in wheat gluten or related proteins from barley, rye, and oats, some of which are considered to be immunodominant (Sollid *et al.*, 2020; Chubnova *et al.*, 2023). Although gluten proteins are clearly dominant in triggering CD, other wheat proteins may also be able to trigger the response in some individuals, notably ATIs (reviewed by Geisslitz *et al*., 2021).

Although the prevalence of wheat allergy is low and the responses are in most cases not life threatening, a rare form called Wheat Dependent Exercise-Induced Anaphylaxis (WDEIA) may result in severe reactions. Gluten proteins (notably monomeric gliadins) and ATIs have also been identified as able to stimulate IgE-mediated allergic responses to wheat in foods (reviewed by Tatham and Shewry, 2008; Brouns *et al*., 2019).

Less is understood about the substances that are able to trigger NCWS. The initial suggestion that the condition is triggered by gluten proteins has not been substantiated and a recent systematic review found no evidence for effects of either ATIs or Fermentable Oligo-, Di- and Monosaccharides and Polyols (FODMAPs), both of which had been suggested as triggers by previous studies (An *et al*., 2025).

### Removal of components that trigger adverse reactions

The importance of wheat gluten in determining the functional properties of flour and dough, and the pervasiveness of wheat (and to a lesser extent barley, rye, and oats) in processed foods, poses challenges for developing food products that do not trigger adverse responses in susceptible individuals.

Coeliac epitopes are present in the vast majority of the individual gliadin and glutenin proteins that form the gluten fraction although they are more abundant, and immunodominant, in some protein types: gliadins more than glutenins, and α-gliadins more than other gliadin types (reviewed by Shewry and Tatham, 2016). The abundances of coeliac epitopes vary little between different types of wheat, with no evidence of greater abundances in modern types of bread wheat compared to older types and ancient wheats (Ribeiro *et al*., 2016). RNA interference (RNAi) and gene editing can be used to down-regulate gluten proteins with coeliac epitopes and remove individual epitopes, respectively (reviewed by Smulders *et al.*, 2023), while mutation breeding has been used to delete genes encoding α-gliadins with immunodominant epitopes (Rottersman *et al*., 2025). RNAi and gene editing have also been used to reduce the contents of ATIs (Camerlengo *et al*., 2020; Kalunke *et al*., 2020).

However, although the reduction or elimination of individual types or groups of gluten proteins and ATIs may not have negative effects, or even have positive effects (Rottersman *et al*., 2025), on the processing properties of grain samples grown for laboratory comparisons, their impact on the yield and quality of grain grown on a commercial scale has not been established. The use of genetic modification and gene editing also has ethical and regulatory implications, although these do not apply to mutation breeding.

Finally, the development, cultivation, and marketing of wheats with modified protein compositions would require strict segregation throughout the wheat production and utilization chain while developing and deploying the modified traits would result in higher costs for breeders, all of which would result in higher costs for consumers.

### Understanding consumer concerns

Public awareness of the relationship between food and health has increased greatly over the past few decades, with both positive and negative outcomes. On the positive side, consumers have a greater awareness of targets for healthy eating (such as ‘5-a-day’ and the ‘Eatwell Plate’ in the UK) and of the positive and negative impacts of individual foods and food components. For example, the targets to eat more fibre and less highly processed, highly refined, and energy-dense foods. However, information on foods and diets has increasingly been sought from the internet and popular media (including celebrity endorsements) rather than health professionals, particularly in relation to avoiding certain types of food.

This is certainly the case for the increasing consumer demand for gluten-free foods. For example, it was reported that about 8% of UK adults who visited a restaurant more than once in 2019 followed a gluten-free diet (Coeliac UK, 2025). This proportion is clearly higher than those who have been diagnosed as requiring a gluten-free diet by qualified medical practitioners, being largely based on self-diagnosis as ‘gluten sensitive’ or the suggestion the avoiding gluten would result in greater well-being.

The impact of expectation on responses is illustrated by a recent randomized, double-blind, placebo-controlled, international, multicentre study of a cohort of 80 volunteers with self-diagnosed NCWS (de Graaf *et al*., 2024). The cohort was divided into two groups of 40 who were told that they would receive either conventional or gluten-free breads. However, each of these groups was then divided into two groups of 20 who received each bread type. Hence, half of those who expected to receive gluten-free bread received conventional bread and vice versa. Patients then recorded their symptoms after consuming two bread-based meals. This study showed that the group that expected to receive gluten and actually received gluten had higher scores for overall gastrointestinal symptoms, abdominal discomfort, and bloating, than the other three groups. This is consistent with other studies which reported that expectancy was more important than actual consumption in determining symptoms in individuals with NCGS (Biesiekierski *et al*., 2013; Ponzo *et al*., 2021).

The increasing avoidance of wheat also has wider implications for health as gluten-free foods may be more highly refined than conventional foods with lower contents of vitamins, minerals, and other essential and beneficial components (Myhrstad *et al*., 2021). Furthermore, it also has implications for sustainability as wheat is one of a few major crops and not only currently feeds a significant proportion of the world population but also has the potential to increase to keep pace with future population increases.

## Conclusion

Much has changed since I contributed a Darwin Review on wheat in 2009 (Shewry, 2009), with greater awareness of threats to environmental sustainability and impacts on health and well-being. But how serious are these threats and do the current concerns about wheat production and consumption have a scientific basis? In this new review I have identified two key challenges.

Firstly, there is no doubt that high levels of nitrogen fertilizer are required to drive high yields. However, this applies to all staple crops as nitrogen is present in all proteins which range in their contents in major crops from about 2% fresh weight (10% dry weight) in potatoes to 36% fresh weight (42% dry weight) in soybean. The nitrogen present in this protein, multiplied by the yield and adjusted for the nitrogen harvest index, therefore represents the minimum amount of nitrogen which must be available to the crop. Although lower yields will require less nitrogen, reducing yields is clearly not realistic if we are to feed the increasing population and the key is to maximize the efficiency of nitrogen application and utilization by the crop, including the nitrogen harvest index.

Secondly, it is important to educate consumers to understand the relationships between wheat, nutrition, and health, and appreciate that wheat should form part of a healthy diet for all except a small proportion of the population with clinically-diagnosed adverse reactions. This will require more effective mechanisms to communicate with consumers as well as increased emphasis on developing types of wheat and processed foods with improved compositions for health.
